# Factors associated with recovery delay in a sample of patients diagnosed by MERS‐CoV rRT‐PCR: A Saudi Arabian multicenter retrospective study

**DOI:** 10.1111/irv.12560

**Published:** 2018-04-25

**Authors:** Anwar E. Ahmed, Hamdan Al‐Jahdali, Mody Alaqeel, Salma S. Siddiq, Hanan A. Alsaab, Ezzeldin A. Sakr, Hamed A. Alyahya, Munzir M. Alandonisi, Alaa T. Subedar, Yosra Z. Ali, Hazza Al Otaibi, Nouf M. Aloudah, Salim Baharoon, Sameera Al Johani, Mohammed G. Alghamdi

**Affiliations:** ^1^ King Abdullah International Medical Research Center (KAIMRC)/King Saud bin Abdulaziz University for Health Sciences (KSAU‐HS)/King Abdulaziz Medical City (KAMC) Ministry of National Guard ‐ Health Affairs Riyadh Saudi Arabia; ^2^ King Fahad General Hospital *‐* Jeddah Jeddah Saudi Arabia; ^3^ Medical Records Department Ministry of Health Jeddah Saudi Arabia; ^4^ King Saud University Riyadh Saudi Arabia

**Keywords:** camel contact, diagnosis delay, MERS‐CoV, PCR, recovery delay

## Abstract

**Background:**

Research evidence exists that poor prognosis is common in Middle East respiratory syndrome coronavirus (MERS‐CoV) patients.

**Objectives:**

This study estimates recovery delay intervals and identifies associated factors in a sample of Saudi Arabian patients admitted for suspected MERS‐CoV and diagnosed by rRT‐PCR assay.

**Methods:**

A multicenter retrospective study was conducted on 829 patients admitted between September 2012 and June 2016 and diagnosed by rRT‐PCR procedures to have MERS‐CoV and non‐MERS‐CoV infection in which 396 achieved recovery. Detailed medical charts were reviewed for each patient who achieved recovery. Time intervals in days were calculated from presentation to the initial rRT‐PCR diagnosis (diagnosis delay) and from the initial rRT‐PCR diagnosis to recovery (recovery delay).

**Results:**

The median recovery delay in our sample was 5 days. According to the multivariate negative binomial model, elderly (age ≥ 65), MERS‐CoV infection, ICU admission, and abnormal radiology findings were associated with longer recovery delay (adjusted relative risk (aRR): 1.741, 2.138, 2.048, and 1.473, respectively). Camel contact and the presence of respiratory symptoms at presentation were associated with a shorter recovery delay (expedited recovery) (aRR: 0.267 and 0.537, respectively). Diagnosis delay is a positive predictor for recovery delay (*r* = .421; *P* = .001).

**Conclusions:**

The study evidence supports that longer recovery delay was seen in patients of older age, MERS‐CoV infection, ICU admission, and abnormal radiology findings. Shorter recovery delay was found in patients who had camel contact and respiratory symptoms at presentation. These findings may help us understand clinical decision making on directing hospital resources toward prompt screening, monitoring, and implementing clinical recovery and treatment strategies.

## 1 INTRODUCTION

Laboratory‐confirmed Middle East respiratory syndrome coronavirus (MERS‐CoV) has been documented in more than 2000 cases worldwide, causing 722 related deaths from September 2012 through September 2017.^1^ Much research evidence is available on factors associated with a poor prognosis in laboratory‐confirmed MERS‐CoV^2^, ^3^, ^4^, ^5^, ^6^, ^7^, ^8^, ^9^, ^10^, ^11^ and non‐MERS‐CoV^9^, ^10^, ^11^ patients. A high mortality rate was systematically recognized in MERS‐CoV patients of old age,^2^, ^12^, ^13^, ^14^, ^15^ severe illness,^2^, ^12^, ^13^, ^14^ underlying condition,^2^, ^12^, ^13^, ^14^ and respiratory/gastrointestinal symptoms.^2^ However, successful management of MERS‐CoV such as clinical recovery and its predictors has not been given sufficient attention despite the virus having been in circulation since 2012.

As per the authors’ knowledge, two studies have so far addressed clinical improvement on laboratory‐confirmed MERS‐CoV patients.^16^, ^17^ The first study, Shalhoub et al,^16^ was based on a case report in which their observations may not be generalized to a wider MERS‐CoV population. The second study, Al‐Turaiki et al,^17^ utilized publicly available data from the Saudi Ministry of Health. The major shortcomings in their study were several potential confounding factors such as underlying medical conditions and a primary or secondary mode of MERS‐CoV transmission that had not been included in the analysis. In addition, the recovery delay was not reported in their study, and thus, factors related to the recovery delay were not examined.

As of October 4, 2017, there was no available detailed data on recovery delay of laboratory‐confirmed MERS‐CoV and non‐MERS‐CoV patients. Data on the time intervals between a patient's presentation or admission to a healthcare facility and the first specimen sample have been limited in patients suspected and screened for MERS‐CoV by a real‐time reverse‐transcription polymerase chain reaction (rRT‐PCR) test, as it might correlate with recovery delay intervals.

Early screening and diagnosis of MERS‐CoV could greatly promote proper control and clinical management of cases, which may reduce the risk of transmission and increase the chance of successful outcomes.^18^, ^19^, ^20^ This study provides more understanding of how long a period (in days) it may take to recover from MERS‐CoV infection. The authors have studied, retrospectively, a cohort of survivors—laboratory‐confirmed MERS‐CoV and non‐MERS‐CoV patients—to estimate recovery delay intervals and identify possible associated factors in Saudi Arabia. The authors assessed whether the time interval between presentation and the initial rRT‐PCR diagnosis (diagnosis delay) correlates with the time interval between initial rRT‐PCR diagnosis and recovery (recovery delay). We hypothesized that older age, MERS‐CoV infection, ICU admission, and abnormal radiology findings might be associated with longer recovery delay. We hypothesized that diagnosis delay might positively correlate with a recovery delay.

## 2 METHODS

A multicenter retrospective study reviewed medical records of 829 patients from September 2012 to June 2016 who were admitted to the hospital and had been diagnosed by rRT‐PCR assay for suspected MERS‐CoV to have MERS‐CoV and non‐MERS‐CoV infection. The study included patients who were admitted through emergency departments (pediatrics and adults) or patients who were admitted through outpatient clinics. Screening referrals for MERS‐CoV was made in accordance with the guidelines set by the Saudi Ministry of Health in standard risk assessment algorithms for identifying and managing MERS‐CoV infection.^21^ In the study population, the rRT‐PCR was used to detect MERS‐CoV in multiple and/or different clinical specimens, including combined nasopharyngeal and throat swabs, sputum, blood, stool, and endotracheal aspirate (ETA).

The study gathered data from the two largest hospitals in Saudi Arabia: King Abdulaziz Medical City in Riyadh (KAMC‐R) and King Fahd General Hospital in Jeddah (KFGH‐JED). Both hospitals, when data were combined, experienced the largest MERS‐CoV outbreak worldwide. The study approval was obtained from two ethical committees in the King Abdullah International Medical Research Center (Study Number: RC17/061) and the Saudi Ministry of Health (IRB Log Number: 16‐230E) in Riyadh Saudi Arabia.

From patient charts, we collected demographic data: age and gender. Elderly age was defined by classifying age into two groups using a cutoff of 65 years (≥65 years). The reason behind this classification was to assess the recovery delay for this vulnerable age group, as a previous study reported a high mortality rate in this group.^15^ We collected data on route of transmission: camel contact and patient contact. The study authors collected various clinical data: fever (temperature ≥ 38°C); presence of any of the following respiratory symptoms: cough, bloody cough, shortness of breath, or chest pain; presence any of the following gastrointestinal symptoms: diarrhea, vomiting, or nausea; MERS‐CoV infection; intensive care unit (ICU) admission; hospital: KAMC‐Riyadh or KFGH‐Jeddah; abnormal radiology findings; diabetes; renal disease; and hypertension.

Recovery delay was calculated as the number of days from the initial rRT‐PCR diagnosis (±), which was the date found on the pathology report of the first specimen, to the clinical recovery (recovery delay), based on the date of hospital discharge or date of MERS‐CoV or non‐MERS‐CoV infection was ruled out. In some cases, the clinical recovery was verified by taking a sample from different types of specimens at varying times. In all patients with initial rRT‐PCR result, the medical records were reviewed from the date of presentation/hospital admission until 60 days after the initial rRT‐PCR diagnosis. Only patients who achieved recovery were analyzed. The study excluded patients with no available clinical recovery records and no discharge records within 60 days after the initial rRT‐PCR diagnosis, as well as patients who had died.

The final sample included 396 laboratory‐confirmed MERS‐CoV and non‐MERS‐CoV patients who had recovered and were identified by reviewing patient charts, hospital discharge records, and medical practitioner notes.

### 2.1 Statistical analysis

The analysis was conducted using IBM Statistical Package for Social Sciences (SPSS) (24; SPSS, Chicago, IL). Patients’ characteristics were described by count and percent, and mean (± standard deviation) or median where appropriate. Time intervals in days from presentation to initial rRT‐PCR diagnosis (diagnosis delay) and from initial rRT‐PCR diagnosis to recovery (recovery delay) were analyzed by Spearman's correlation coefficient.

The Poisson and negative binomial models were used to model the frequency of recovery delay in days and identify unadjusted and adjusted factors associated with longer recovery delay. Goodness‐of‐fit measures were used to compare and identify the best model. The model with the smaller deviance, larger Log likelihood, smaller Akaike information criterion, and smaller Bayesian information criterion was considered the better model. In all analyses, a *P*‐value of less than 5% was considered significant. Relative risk (RR) and 95% confidence intervals (CI) were used to assess the strength of association between patients’ characteristics and longer/shorter recovery delay.

## 3 RESULTS

A total of 396 patients, suspected and screened for MERS‐CoV by an rRT‐PCR test, were analyzed. The average age was 46 years with age ranges between 1 and 95 years. The median recovery delay in our sample was 5 days. Of the sample, 57.7% were male and 18.4% were admitted to ICU. Fever and respiratory symptoms were common presentations, occurring in 66.3% and 84.1% of the patients, respectively. The chest X‐ray and/or CT scan were abnormal in almost half of the samples (48.4%). Refer to Table 1 for other sample parameters. The longer delays in diagnosis were positively correlated with longer recovery delay (*r* = .421; *P* = .001).

**Table 1:** Distribution of sample characteristics (N = 396)

Characteristics	Levels	n	%
Elderly	Yes	117	29.6
	No	278	70.4
Gender	Female	167	42.3
	Male	228	57.7
Patient contact	Yes	21	5.3
	No	375	94.7
Camel exposure	Yes	6	1.5
	No	314	79.3
	Unknown	76	19.2
MERS‐CoV infection	Yes	53	13.4
	No	343	86.6
ICU admission	Yes	73	18.4
	No	323	81.6
Hospital	KAMC‐Riyadh	270	68.2
	KFGH‐JEDDAH	126	31.8
Fever	Yes	262	66.3
	No	133	33.7
Respiratory symptoms	Yes	333	84.1
	No	63	15.9
Gastrointestinal symptoms	Yes	97	24.5
	No	299	75.5
Abnormal radiology findings	Yes	169	48.4
	No	180	51.6
Diabetes	Yes	143	36.1
	No	253	63.9
Renal disease	Yes	44	11.1
	No	352	88.9
Hypertension	Yes	143	36.1
	No	253	63.9

MERS‐CoV, Middle East respiratory syndrome coronavirus; KAMC‐Riyadh, King Abdulaziz Medical City in Riyadh; KFGH‐JEDDAH, King Fahd General Hospital in Jeddah

According to univariate negative binomial regression analysis (Table 2), shorter recovery delay was noted in patients with camel contact (relative risk (RR) = 0.134; 95% CI: 0.045‐0.398). The univariate analysis showed longer recovery delay in elderly patients (65 years or over) (RR = 1.343; 95% CI: 1.069‐1.686), patients with MERS‐CoV infection (RR = 2.556; 95% CI: 1.895‐3.447), ICU patients (RR = 2.915; 95% CI: 2.239‐3.794), patients with abnormal radiology findings (RR = 2.016; 95% CI: 1.612‐2.521), patients with diabetes (RR = 1.356; 95% CI: 1.092‐1.683), patients with renal disease (RR = 1.454; 95% CI: 1.048‐2.018), and patients with hypertension (RR = 1.440; 95% CI: 1.160‐1.788).

**Table 2:** Factors associated with recovery delay in a sample of patients diagnosed by rRT‐PCR (N = 396)

Characteristics	Reference	Univariate negative binomial regression				Multivariate negative binomial regression			
		*P*	RR	95% CI for RR		P	aRR	95% CI for RR	
				Lower	Upper			Lower	Upper
Elderly (65 y)	65 y	.011^a^	1.343	1.069	1.686	.001^a^	1.741	1.276	2.374
Female	Male	.996	1.001	0.810	1.236	.424	1.103	0.868	1.402
Patient contact	No	.881	0.965	0.605	1.539	.683	0.886	0.496	1.584
Camel exposure	Unknown	.001^a^	0.134	0.045	0.398	.026^a^	0.267	0.083	0.855
No camel exposure	Unknown	.077	0.788	0.606	1.026	.783	1.053	0.729	1.521
MERS‐CoV infection	No	.001^a^	2.556	1.895	3.447	.001^a^	2.138	1.378	3.318
ICU admission	No	.001^a^	2.915	2.239	3.794	.001^a^	2.048	1.450	2.892
Hospital: KAMC‐Riyadh	KFGH‐Jeddah	.605	1.061	0.848	1.328	.042^a^	0.696	0.491	0.987
Fever	No	.248	0.878	0.704	1.095	.326	0.879	0.679	1.137
Respiratory symptoms	No	.124	0.801	0.603	1.063	.001^a^	0.537	0.387	0.745
Gastrointestinal symptoms	No	.054	0.786	0.616	1.004	.342	0.866	0.643	1.166
Abnormal radiology findings	No	.001^a^	2.016	1.612	2.521	.003^a^	1.473	1.144	1.896
Diabetes	No	.006^a^	1.356	1.092	1.683	.664	0.925	0.652	1.314
Renal disease	No	.025^a^	1.454	1.048	2.018	.061	1.412	0.985	2.025
Hypertension	No	.001^a^	1.440	1.160	1.788	.358	1.175	0.833	1.658

CI, confidence interval; RR, relative risk; aRR, adjusted relative risk.

a Significant at α = 0.05.

**Figure 1:**
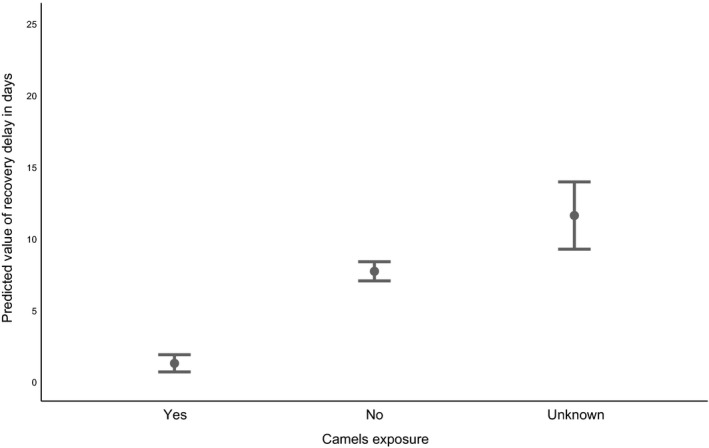
The impact of camel exposure on recovery delay

**Figure 2:**
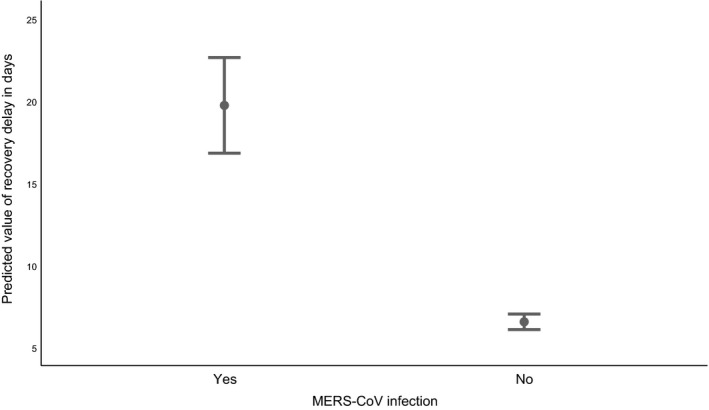
The impact of MERS‐CoV infection on recovery delay

**Figure 3:**
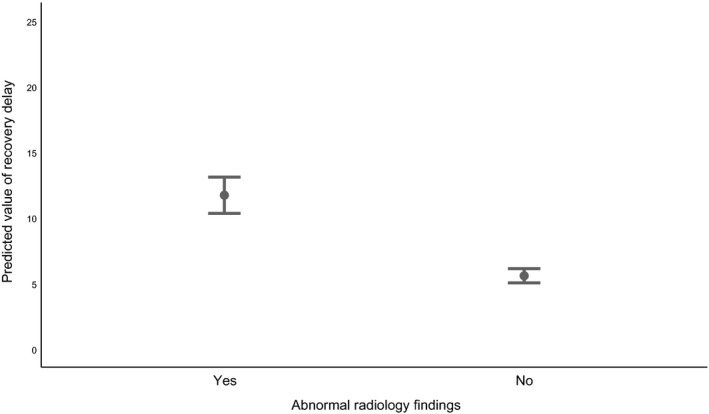
The impact of abnormal radiology findings on recovery delay

A multivariate negative binomial regression analysis (Table 2) revealed six independent factors that affect the recovery delay. Camel contact (adjusted relative risk (aRR)  = 0.267; 95% CI: 0.083‐0.855) (Figure 1) and respiratory symptoms (RR = 0.537; 95% CI: 0.387‐0.745) were major independent factors associated with shorter recovery delay. Elderly (65 years or over) (RR = 1.741; 95% CI: 1.276‐2.374), MERS‐CoV infection (RR = 2.138; 95% CI: 1.378‐3.318) (Figure 2), ICU admission (RR = 2.048; 95% CI: 1.450‐2.892), and abnormal radiology findings (RR = 1.473; 95% CI: 1.144‐1.896) (Figure 3) were associated with longer recovery delay.

We compared goodness‐of‐fit measures between the negative binomial and the Poisson models. The goodness‐of‐fit measures indicate that the negative binomial model fits the data better than the Poisson model. The negative binomial model had smaller deviance (1.05 vs. 6.70), larger Log likelihood (−1031.54 vs. −1649.60), smaller Akaike information criterion (2095.08 vs. 3331.19), and smaller Bayesian information criterion (2156.66 vs. 3392.78) than the Poisson model.

## 4 DISCUSSION

This is the first study identifying predictors of recovery delay (in days) in a large sample of laboratory‐confirmed MERS‐CoV and non‐MERS‐CoV patients. Despite recovery delay being an important indicator of MERS‐CoV infection progression, it remains poorly understood in this population. The data were collected from two of the largest tertiary hospitals in Saudi Arabia: KAMC‐R and KFGH‐JED from September 2012 to June 2016.

According to our research, evidence suggests that there are a number of factors that affect the course of recovery delay in suspected MERS‐CoV patients whose clinical samples were evaluated by the rRT‐PCR test. Older age (65 years or over) was a major predictor of longer recovery delay in our sample. This was noted by Al‐Turaiki et al, as well.^17^ In other recent studies, being of older age was a factor for worse clinical outcomes such as infection severity^13^ and death^2^, ^13^, ^14^ in MERS‐CoV patients. This age group has been linked to a number of pre‐existing medical conditions and other health risks which can also increase the risk of longer recovery delay in this population. It is essential that the healthcare practitioners who provide direct medical care to suspected MERS‐CoV patients most carefully monitor infection development to avoid poor outcomes in elderly patients.

As expected, the risk of longer recovery delay was twice as high in patients with MERS‐CoV infection than patients without MERS‐CoV infection. This finding could be attributed to several factors. MERS‐CoV is a serious illness and is very common in patients in the older age group^2^, ^12^, ^13^, ^14^, ^15^ and patients with pre‐existing medical conditions,^2^, ^11^, ^12^, ^13^, ^14^ and these seem to increase the risk of early mortality after diagnosis.^2^ Furthermore, most MERS‐CoV patients develop severe pneumonia^22^ and necessitate mechanical ventilation,^11^ which is a risk factor for hospital mortality.^23^

Patients admitted to ICU admission were at higher risk for longer recovery delay. A previous report showing similar findings, longer ICU stay, and high mortality rate was reported in MERS‐CoV patients who were admitted to the ICU.^24^ Such patients would benefit from monitoring their responses to medical support and recognizing potential complications at an early stage.

We found that camel contact was associated with shorter recovery delay. Studies on recovery delay in patients with camel contact as compared to close contact exposure of a confirmed case or other exposure are lacking; however, camel contact has also been linked to lower 3‐ and 30‐day mortality rates in MERS‐CoV patients.^2^ This important association requires more studies to identify whether camel contact is an independent protective factor of shorter recovery delay.

In our study, patients with respiratory symptoms are more likely to experience shorter recovery delay than patients without respiratory symptoms. This finding is probably related to the shorter lag time between symptom onset and diagnosis, in which patients with presence of symptoms could be positively affected by an early diagnosis^18^ and thus prompt medical support is deemed necessary.

The time interval from presentation to initial rRT‐PCR diagnosis (diagnosis delay) was positively correlated with the time interval from initial rRT‐PCR diagnosis to recovery (recovery delay). Early diagnosis is likely to improve clinical outcomes^18^ and reduce the economic and physical burden of a disease.^25^, ^26^ Early diagnosis requires full utilization of hospital resources. Individuals at high risk of MERS‐CoV infection should be promptly screened after arrival at the healthcare facility, monitored for progression, and then having a prompt decision made for whether further rRT‐PCR testing is needed.

The authors noticed the following limitations. First, the study was based on chart reviews, and findings should be interpreted with caution. Second, we did not collect information on the type of antiviral treatment or other supportive treatments given after diagnosis which may have affected clinical outcomes.^27^, ^28^ Third, despite this being the first investigation in this population, including a number of potential predictors for recovery delay, additional relevant predictors should be explored, such as the level of camel exposure, for example, hospital‐acquired infections. Fourth, patients with clinical recovery were identified by reviewing the medical records of the study sample within 60 days after the initial rRT‐PCR diagnosis. Studies with longer periods of follow‐up in a larger population recovering from MERS‐CoV are warranted to assess the long‐term successful clinical outcomes.

Despite the mentioned limitations, data were aggregated directly from medical charts rather than public source databases. This chart review study was based on information from multicenters and a large sample size, and it provides valuable information on factors associated with prolonged or shorter recovery delay of patients suspected and screened for MERS‐CoV by the rRT‐PCR test. It is essential to develop interventional programs or guidelines to ensure early diagnosis, as this may reduce recovery delay intervals as well as improve patients’ clinical outcomes. This research may enable identification of patients who require receiving appropriate medical support and care according to their illness progression. This also may prevent spread and transmission of the infection as individuals who are still severely ill can be appropriately isolated and managed apart from others who are responding to medical care.

## 5 CONCLUSIONS

The study evidence supports that longer recovery delay was seen in patients with older age, MERS‐CoV infection, ICU admission, and abnormal radiology findings in a sample of patients diagnosed by rRT‐PCR. Recovery delay was significantly shorter in patients who had camel contact and respiratory symptoms at presentation. A prospective study is needed to evaluate the impact of camel exposure on recovery. Evidence was found of an increasing recovery delay with a longer diagnosis delay. The findings may help understand clinical decision making as it directs hospital resources toward prompt screening, monitoring, and implementing clinical recovery and treatment strategies.

## CONFLICT OF INTEREST

There are no conflict of interests.
